# Implementing no-signaling correlations as a service

**DOI:** 10.1038/s41598-024-59492-8

**Published:** 2024-05-10

**Authors:** Mátyás Koniorczyk, Péter Naszvadi, András Bodor, Ottó Hanyecz, Peter Adam, Miklós Pintér

**Affiliations:** 1 Department of Quantum Optics and Quantum Information, Institute for Solid State Physics and Optics, HUN-REN Wigner Research Centre for Physics, Budapest, Hungary; 2 Faculty of Informatics, Eötvös Loránd University, Budapest, Hungary; 3 Institute of Physics, University of Pécs, Pecs, Hungary; 4 Corvinus Center for Operational Research, Institute of Advanced Studies, Corvinus University of Budapest, Budapest, Hungary

**Keywords:** No-signaling correlations, Quantum nonlocality, Cryptographic protocols, Quantum information, Software

## Abstract

We deal with no-signaling correlations that include Bell-type quantum nonlocality. We consider a logical implementation using a trusted central server with encrypted connections to clients. We show that in this way it is possible to implement two-party no-signaling correlations in an asynchronous manner. While from the point of view of physics our approach can be considered as the computer emulation of the results of measurements on entangled particles, from the software engineering point of view it introduces a primitive in communication protocols that can be capable of coordinating agents without revealing the details of their actions. We present an actual implementation in the form of a Web-based application programming interface (RESTful Web API). We demonstrate the use of the API via the simple implementation of the Clauser–Horne–Shimony–Holt game.

## Introduction

The study of nonclassical correlations was triggered by the Einstein–Podolsky–Rosen paradox^1^ raising a fundamental question of physics. The problem was first quantified by Bell^2^ who studied a scenario of two separated parties are in hold of a physical system each, so that the two systems had interacted before. He pointed out that if the parties can choose between different measurements on their systems, the measurement results can show correlations that cannot be explained by the assumption of pre-shared randomness; this is reflected in the violation of certain inequalities. The underlying physical phenomenon is quantum entanglement^3,4^. Notably, the correlations obey the no-signaling property: they cannot be used for transmitting information between the parties. The first experiment to verify such correlations was proposed by Clauser, Horne, Shimony, and Holt ^5^, however, it was an extremely hard task to produce such correlations with the technology of the 1960s.

The evolution of lasers and nonlinear optics in the 1990’s, notably the availability of entangled photon pairs^6^ has brought Bell-type correlations to the forefront of research interest. The structure of quantum and generic nonlocal no-signaling correlations has been broadly studied and understood^7^. Device-independent quantum cryptography^8,9^, based on this kind of correlations, is now one of the most promising technologies, and a broad variety of protocols have been designed and demonstrated for numerous tasks, including secure key distribution^10^, bit commitment^11^, or digital signatures^12^. Quantum communication with satellites became now reality^13^, and quantum communication networks are being built^14^.

Even though nonlocal no-signaling correlations have been discovered with motivations dominantly rooted in Physics, they are of interest per se, also in other scientific fields^15^. From a system engineering point of view, one can think of protocols in which there are connections between parties that do not facilitate communication but can coordinate actions of the parties. This can be relevant even when the formation of these correlations is not instantaneous and their implementation is carried out via the communication with a trusted server on encrypted channels. This is the approach we follow in the present paper: the nonlocal correlations are generated by allowing the software components that implement them to communicate with a central trusted server. We call this “logical implementation”, as opposed to “physical implementations” based on quantum measurements.

It is important to note that our implementation assumes the existence of a communication channel between the nodes and a central server, hence, the direct communication between the parties cannot be excluded by the laws of physics, unlike in the case of physical implementations. If, however, the parties’ activity is restricted to use the implemented no-signaling correlations, these alone do not allow for any communication. Certainly this approach excludes applications aiming at the creation of encrypted channels, like quantum key distribution, however, there are many other possible applications to discover. Game theory^16,17^ can serve as a guideline for designing such applications, in which the coordination without sharing local details is important. Supra-quantum no-signaling correlations, that is, those which cannot even be realized using quantum systems without interaction, are also important in theory^18^, and their possible applications. In the lack of an accessible implementation such applications have been hitherto largely unexplored.

As for the technical implementation, our service relies on RESTful WEB API technology; the dominant one in network services currently. A software library can be easily developed in virtually any development environment or programming language that hides the otherwise simple details of low-level API operation. This facilitates the implementation, development, and testing of any protocol based on nonlocal no-signaling correlations, the development of computer applications using such resources, etc. This can be useful in the better understanding of actual experiments^10^ or optimization of protocols^19^.

From the point of view of physics, a logical implementation is a computer emulation of quantum correlation experiments or protocols, that, unlike physical implementations, requires interaction between parties and the formation of correlations is not instantaneous. However, the aforementioned library can be easily modified to use a physical device’s API instead of the web-service based emulation. Recall that the ETSI standards for quantum key distribution have also resulted in a RESTful API specification^20^, and it has been an important step in the standardization of QKD technology to establish its specification, making quantum key distribution accessible for system engineers.

It is likely that if the quantum technology to physically implement certain nonlocal no-signaling correlations will mature, the physical devices will practically appear in a way similar to our present implementation to a software developer. In this way an application developed using our framework can be easily modified to use physical hardware in the future as quantum communication devices become prevalent and affordable. Currently, on the other hand, it enables the development and testing of protocols without the need of the currently costly or not-yet-existent devices which can be readily converted to use new physical hardware as soon as it becomes actually available.

Beside the system engineering aspects, the implementation results in a deeper understanding of no-signaling correlations, especially their asynchronous nature which is not frequently mentioned. While asynchronous nature is a straightforward consequence of the no-signaling principle, the no-signaling condition is essential for our particular implementation to work. In other words, the implementation of signaling correlations requires a different protocol. This aspect has motivated us in the discovery of the first such protocol in which the parties have to use their no-signaling resources in different order^21^.

This paper is organized as follows. First we provide a brief introduction to the theory of no-signaling boxes. Then we describe our result which is the introduction of the notion of a “logical implementation”, and the algorithm that realizes it. We then describe the methodology: the system architecture and the key details of implementation. Then we discuss a particular example in detail, which showcases our approach in action. This also demonstrates the use of nonlocal no-signaling correlations as a service in a protocol engineering scenario. Finally the results are summarized and conclusions are drawn.

## No-signaling boxes

Consider two parties, Alice and Bob, who are physically separated from each other so the communication between them is excluded, apart possibly from the following. They have access to a device (or, more precisely, a pair of devices) which generates pairs of random variates $(a,b)\in \mathcalligra {A}\times \mathcalligra {B}$ so that the variate *a* is available only for Alice while the other variate *b* is available only for Bob. Each output pair depends on an input pair, too, so that Alice’s input $x\in \mathcalligra {X}$ is entered by Alice locally, and so is Bob’s $y\in \mathcalligra {Y}$. The distribution of the output variates depends on the pair of (local) inputs $(x,y)\in \mathcalligra {X} \times \mathcalligra {Y}$ according to the conditional probability distribution *P*(*a*, *b*|*x*, *y*). Such devices will be termed as a “pairs of boxes”, or simply a “box” in what follows. We will assume the sets $\mathcalligra {A}$, $\mathcalligra {B}$, $\mathcalligra {X}$, $\mathcalligra {Y}$ to be finite. So far we allow for arbitrary correlations; many bipartite boxes would enable communication between the parties, though we will focus on those which do not in what follows.

When a box is used multiple times the input pairs and the corresponding variate pairs have to be labeled. The labels *k* will be elements of an arbitrary index set $\mathcalligra K$, and the tuple $(a_k,b_k,x_k,y_k)$ will be termed as the data of *transaction* *k*. (We note here that in some contributions a given transaction, i.e. a single use of a box is referred to as an instance of a box. When compared to those works, a “box” there is a “transaction” in our terminology.) We do not prescribe any ordering on the set $\mathcalligra {K}$, albeit in practical realizations it is frequently related to time, e.g. due to a causal ordering. We assume that the probability distribution of the variates $(a_k,b_k)$ in a given transaction *k* is entirely determined by $x_k$, $y_k$, and *P*(*a*, *b*|*x*, *y*), and is independent from any other inputs or outputs in the other transactions.

Let us now restrict our attention to those boxes, which cannot be used for the parties to communicate. This is the exclusion of signaling: it implies that Alice and Bob cannot use their box to implement a communication channel solely by using the boxes. In mathematical terms this can be expressed with the following no-signaling conditions:

1 $$\begin{aligned} \sum _b P(a,b|x,y) = P(a|x)\quad \forall y, \end{aligned}$$

and similarly

2 $$\begin{aligned} \sum _a P(a,b|x,y) = P(b|y)\quad \forall x. \end{aligned}$$

Note that these conditions imply the existence of local marginals of the joint conditional probability distribution. Hence, it is possible to operate the boxes asynchronously: Alice can provide $x_k$ anytime, obtaining $a_k$ immediately, and the same holds for Bob, $y_k$ and $b_k$. The times of when a party use the box in a given transaction, and thus the order of the uses is independent. This property can also give rise to interesting protocols ^21^. In what follows we will restrict ourselves to no-signaling boxes.

**Figure 1:**
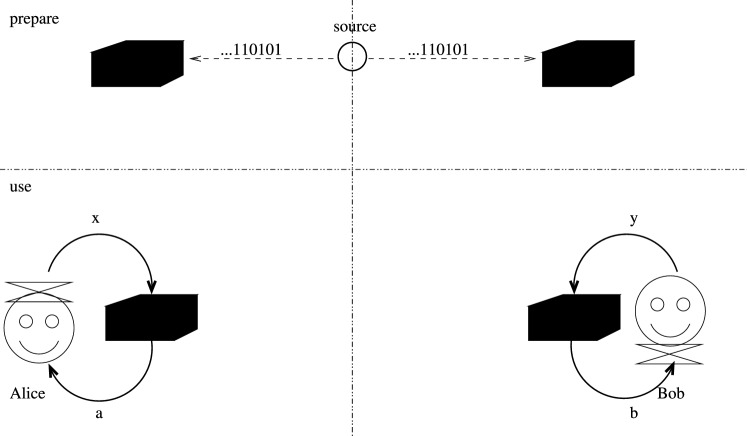
A local box pair: it can be implemented with randomness shared in advance. The vertical dash-dotted line represents spatial separation, whereas the horizontal one represent difference in time. Thus there are two phases: the preparation of the box and the actual use. In the second phase no communication is allowed between the boxes.

The notion of locality of a box pair is to consider those which can be realized with randomness shared in advance before the transaction. A scenario with such a box pair is illustrated in Fig. 1.

Such boxes are described by conditional probability distributions that can be expressed as a convex combination of products of local deterministic boxes. A local deterministic box on Alice’s side assigns a given *a*(*x*) to each *x*, whereas such a box at Bob’s side assigns a given *b*(*y*) to each *y*; their product is the parallel application of the two. Such a pair of boxes has a deterministic (Dirac) conditional probability distribution. Randomness shared in advance enables the realization of any convex combination of these distributions without any communication between the parties. Such boxes are termed as “local”.

**Figure 2:**
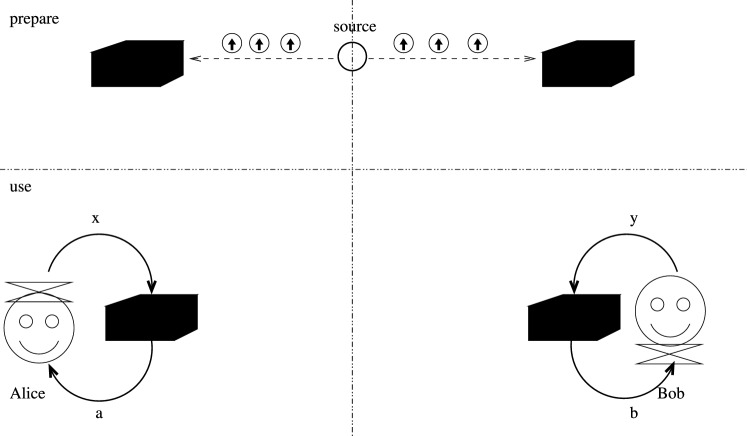
A quantum box pair, compare also with Fig. 1. The circles with upwards arrows inside represent quantum systems (e.g. particles); they are shared in advance. Due to their interaction at the source, they form pairs which are entangled. This enables the realization of nonlocal no-signaling correlation, albeit not the most general ones.

No-signaling boxes form a significantly larger set than that of the local boxes. Therefore there exist “nonlocal correlations” which are interesting both fundamentally and in applications. Some of these can be realized with physical arrangements (i.e. quantum mechanically) in such a way that there is no interaction needed between the parties when using the boxes. In such implementations, however, pairs of quantum systems in entangled state is to be shared in advance, similarly to the pre-shared randomness in the case of local boxes. This scenario is depicted in Fig. 2.

The physical systems are particles; typically photons in case of many realizations. The parts of the system are initially at the same location, a source, and they are interacting, which results in an entangled state in some of their internal degrees of freedom, like the polarization of the two photons. The two subsystems are then sent to the parties Alice and Bob, who choose the measurements corresponding to the inputs *x* and *y* and carry them out on their particle to get *a* and *b* as the measurement result.

Notably, the operation is instantaneous: the parties obtain the (correlated) results immediately after sending the input to the box on both sides, even if the parties’ separation is space-like, and thus there is no way to communicate. Whenever Alice and Bob carry out their measurements, the results are readily available right after the completion of the measurement of each party; there is no need to wait the minimum time that would allow the two sites to communicate. (Recall that information can only be propagated at a limited speed. There will be a “local” reaction time of the box, but this can be negligible.) This feature can be important in certain applications^16^.

The measurement by each party is done solely on the particle available to the given party. Thus there is no interaction or communication between the boxes at the parties (after sharing the pair of particles). Nor there is any interaction or communication between the boxes of Alice and Bob. Thus it is guaranteed that no other party will know about the particular values of *x*(*y*) and *a*(*b*) but Alice (Bob).

Note also that from the no-signaling principle it follows that the two parties may carry out their respective measurements anytime, in arbitrary causal order, without synchronization. If Alice and Bob could store the particles for an arbitrarily long time, they could share enough entangled particle pairs in advance and they could choose freely when to make a given measurement. In practice, however, the coherence times of such particles is short, thus the entangled state is destroyed within a very short time. Hence, in practical scenarios they obtain the particles from a central source (e.g. via fibers or free-space propagation), and often there is a time synchronization to ensure that the measurements are associated with actual members of pairs. Hence, the realization of arbitrary timings, that is, using up pairs with different timing and ordering deliberately on the two sides has up to our knowledge not yet been explored in experiments, although it would not be impossible, apart from some challenges due to loss and decoherence.

Boxes that can be realized physically include local boxes as a proper subset, and they are a proper subset of no-signaling boxes. The structure of the set of physically realizable boxes is defined by the laws of quantum mechanics, we will not go into detail but will show an example of this kind. Our logical implementation covers nonlocal no-signaling boxes in general.

## Results

Our goal is to logically implement a pair of nonlocal no-signaling boxes whose behavior is described by a given conditional probability distribution *P*(*a*, *b*|*x*, *y*), so that it is accessible from software applications. From the point of view of quantum nonlocality, the logical implementation is a computer emulation of the behavior observed in the experiment. We define first what we mean by a logical implementation or emulation as opposed to the physical realizations. Then we describe the principle of the actual algorithm.

### Logical implementation

**Figure 3:**
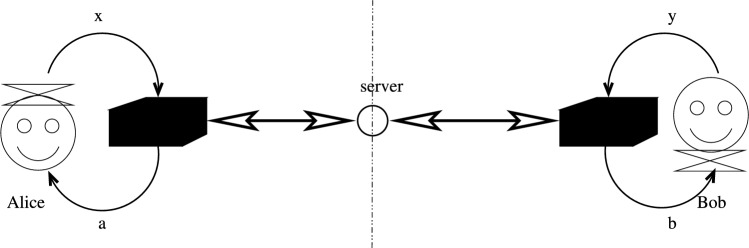
The nonlocal box emulation scenario. A bidirectional communication is allowed between *the boxes* of Alice and Bob, via an encrypted channel, with a trusted server, during the whole process of using the box. Meanwhile Alice and Bob are still not able to communicate each other by using the box.

In our scenario we accept that there is two-way communication between *the boxes* at the parties and a trusted server. This is depicted in Fig. 3.

We require, however, that while the boxes themselves communicate, the parties cannot use the box pair for sending any information: the correlations are no-signaling from the actors’ perspective. Otherwise speaking, the *correlations* themselves are nonlocal, regardless of the implementation.

When comparing with the physical implementation, as a trivial consequence of being generated via communication the central server will have all information about the results, and also there is a need to wait for the completion of the communication with the central server before the result becomes known, so the formation of the correlations is certainly not instantaneous. On the other hand, because of the no-signaling principle, no synchronization assumed and the set of the transactions $\mathcalligra {K}$ does not need to have a causal structure. As we will point out later, this feature is easily implemented in this framework.

As an additional benefit, it is certainly possible to implement supra-quantum correlations; those which are no-signaling but cannot be realized quantum mechanically, such as a Popescu–Rohrlich (PR) box that will be described in detail later. Assuming that the server is trusted, the communication between the server and the boxes is secure, and that the parties use the boxes according to the prescription, such an implementation can be interesting *per se*. We conclude this subsection with tabulating the required resources and the features of the various implementations in Tables 1 and 2.

**Table 1:** A comparison of resources required to realize a local, a quantum, and a logically implemented generic no-signaling box pair.

Type	Local	Quantum	Logical
Shared randomness	Yes	No	No
Entanglement	No	Yes	No
Bidirectional secure communication	No	No	Yes

**Table 2:** A comparison of features offered by a local, a quantum, and a logical (emulated) no-signaling box pair.

Type	Local	Quantum	Logical
Instantaneous	Yes	Yes	No
Interaction-free	Yes	Yes	No
Quantum confidential	Yes	Yes	No
Quantum behaviors	No	Yes	Yes
Supra-quantum behaviors	No	No	Yes

### Algorithm

**Figure 4:**
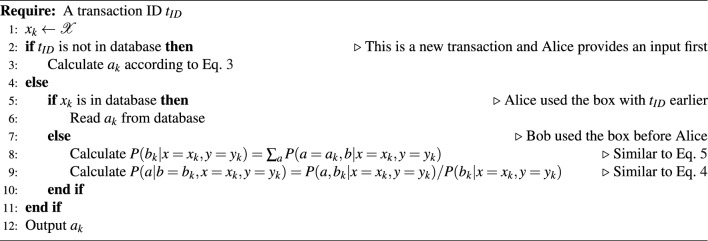
The pseudocode of the API call useBox on Alice’s side. Let $x_k$ ($y_k$) denote Alice’s (Bob’s) input. If Bob is the first, then the roles are reverted.

Let us now describe the actual algorithm of our implementation. A pseudocode for the algorithm is provided in Fig. 4. Assume first that Alice is the first to send her input, that is, she uses her box with the given transaction in time before Bob. (Recall that no synchronization is assumed but the transaction is uniquely identified by a value of *k*.) So Alice sends a particular $x_k$ value in transaction *k* to the box. The result $a_k$ of the box is drawn according to the local marginal

3 $$\begin{aligned} P(a|x=x_k)=\sum _b P(a,b|x=x_k,y=\overline{y}) \end{aligned}$$

where $\overline{y}\in \mathcalligra {Y}$ is an arbitrary fixed *y* (due to the no-signaling condition in Eq. (1) any element can be chosen). The respective value of the random variate $x_k$ is sent to Alice, while the triple (*k*, *x*, *a*) is stored in the database.

If Bob provides his input $(k,y_k)$ later and asks for his output $b_k$, it is a random variate drawn according to the conditional distribution

4 $$\begin{aligned} P(b|a=a_k,x=x_k,y=y_k)=\frac{P(b,a_k|x=x_k,y=y_k)}{P(a_k|x=x_k,y=y_k)}, \end{aligned}$$

where

5 $$\begin{aligned} P(a_k|x=x_k,y=y_k) = \sum _b P(b,a=a_k|x=x_k,y=y_k). \end{aligned}$$

and the transaction is completed (after storing all details in the database). As the protocol is symmetric, when Bob is the first to initiate transaction *k*, the roles are reverted but the procedure is the same.

In a software implementation it is therefore vital to ensure the following condition. When transaction *k* has been initiated by Alice, no reply to Bob can be generated before the transaction has concluded for Alice, that is, before *a* is generated and $(k,x_k,a_k)$ has been stored. The same holds for Bob’s initiation of transaction *k* for $(k,y_k,b_k)$. Using conventional relation database management, this can be ensured by locking the table of transactions, or at least transaction *k* whenever it is acted upon on behalf of either of the parties.

Note that there can be two kinds of actions: if the transaction was already initiated by the other party then we use the joint probability with the known condition, whereas if it wasn’t we just use the local marginal but keep the given input. Looking at the empirical marginals ex post, they will follow the local marginal distributions that exist because of the no-signaling condition.

## Methodology

**Figure 5:**
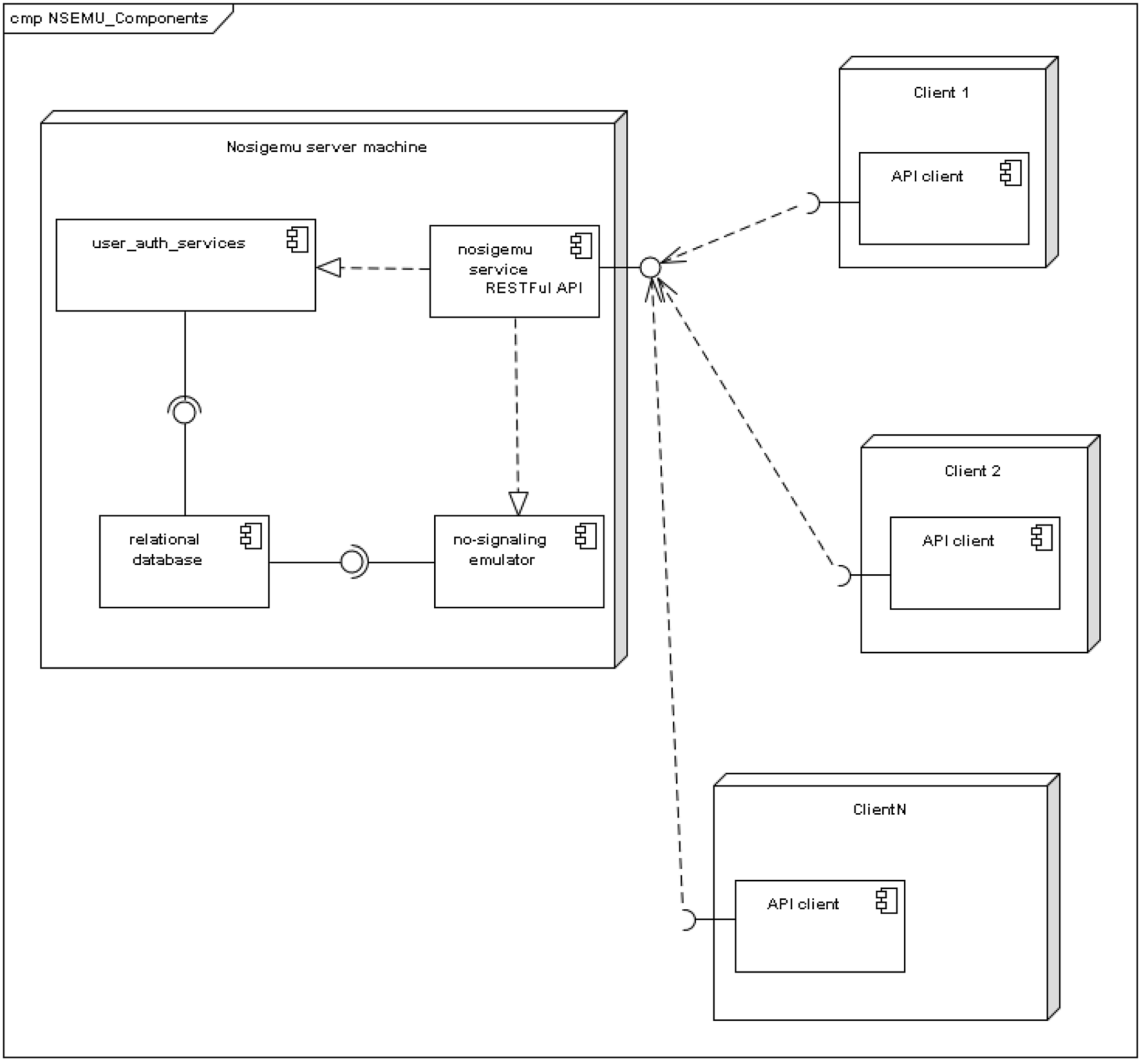
UML^22^ component diagram of the software architecture.

In this Section we describe the software architecture that has been used for the implementation. The components of the IT architecture are depicted in Fig. 5. The implementation is based on a central service run on a server. The service provides a RESTful API to clients, using HTTP GET requests with URL parameters, and returning the result in JSON format. (An example of a session will be presented later.)

The server component realizes a component needed for user authentication and management, and a component that realizes the box emulator algorithm. Both of the components use the same underlying relational database which they communicate via its standard internal interface.

The server component is implemented in Python programming language. It is based on SQLAlchemy ^23^ as an object-relational mapper and Flask ^24^ as the WEB API provider framework. The currently running beta version uses PostgreSQL ^25^ as a relational database manager. The random variates used by the server at the time of writing this paper are obtained from a “Quantis” USB Quantum Random Number Generator, model “USB-4M”, manufactured by “ID Quantique”^26^ with the serial number 184443A410. The Python library for accessing this device was also developed in the framework of the present project^27^. At the time of the publication of this article as an e-print, the beta version will be available for the public after a free registration, for academic and educational purposes ^28^.

**Figure 6:**
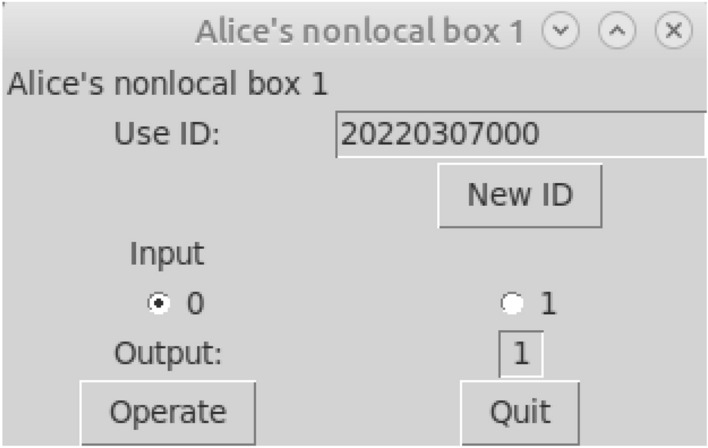
A simple desktop graphical user interface for the logical nonlocal box implementation.

Owing to the use of a standard API, a client can be any device running any software that is capable of consuming RESTful APIs at a basic level. Hence the possible client implementations and devices range from tutorial codes in various programming languages through smartphone applications to test cryptographic protocol implementations. A screenshot of a simple desktop graphical user interface is to be found in Fig. 6.

## Discussion

In this section we demonstrate the use of the no-signaling correlations API by using it to implement the so-called Clauser–Horne–Shimony–Holt (CHSH) game ^5,17^. It can be considered as a tutorial project with pairs of participants who implement their box pair, and then play the game with and without using it. It also illustrates the possible role of the service in the design of a communication protocol.

The game itself is the following. The two players, Alice and Bob are separated and are not allowed to communicate. In each turn of the game Alice randomly chooses an input *x* which is 0 or 1, while Bob randomly chooses an input *y* which is 0 or 1. Importantly, they have to be really honest about choosing these with a uniform distribution. Alternatively they can be provided these inputs by a trusted source. Then Alice says an output *a*, Bob says an output *b*. They both get a unit of reward in the following two cases: if both of them chose 1 as input ($x=y=1$), and their output is the opposite, i.e. $a=1$, $b=0$ or $a=0$, $b=1$, or if any of them had 0 as an input, and their outputs *a* and *b* are same. Otherwise there is a unit of negative payoff. The payoff function is thus the same for both parties; it is tabulated in Table 3.

**Table 3:**
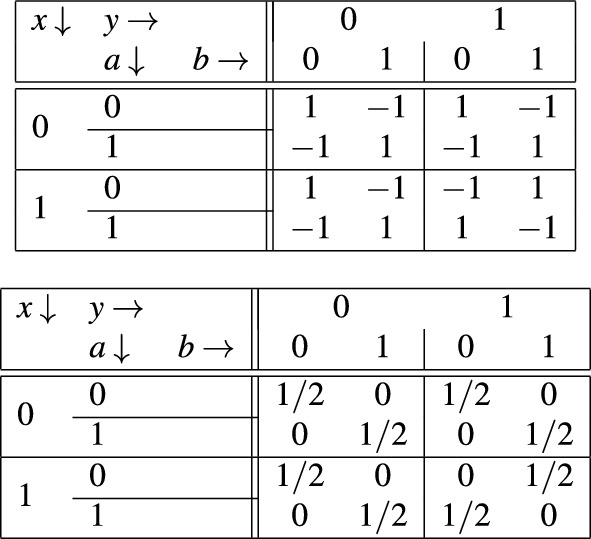
The payoff function of the CHSH game (top) and the PR-box, the no-signaling behavior maximizing it (bottom).

This game is the so-called Clauser–Horne–Shimony–Holt (CHSH) game, which is behind the celebrated CHSH inequality. It can be proven that if Alice and Bob are not allowed to communicate and are restricted to use pre-shared randomness (even an infinite sequence of correlated random bits, shared before their separation), the best they can do is the following: they agree in advance to always say $a=0$ ($b=0$) regardless of their inputs *x* and *y*. In that case they will win in 75% of the cases, that is, in case of uniformly distributed input pairs they get 1 in 3/4 of the cases and -1 in 1/4 of them, so the average payoff will be 1/2. It can be proven that no other, even randomized strategy involving pre-shared randomness can result in a better payoff. This limit on the payoff is the Bell-CHSH inequality.

The limit of 1/2 on the average payoff can be overcome when the parties are allowed to use a pair of no-signaling boxes. The nonlocal no-signaling behavior of a box pair that enable Alice and Bob to obtain the maximal average payoff of 1, is also tabulated in Table 3; is called the Popescu–Rohrlich box. If they both feed their box with their inputs *x* and *y* and they provide the respective output *a* and *b* as the output, they will be positively rewarded in all the cases. It can be shown, however, that the access to such a box pair does not enable to send any message or signal to the other. They get, however, coordinated without communication.

Let us now see how the game-play is actually implemented using the API calls. (We will use the  curl  command available on most Linux systems to communicate the API with GET request. Alternatively, the URL can be written into the browser.)

1. Alice sends $x=0$ as her input to box 1, a PR-box. The transaction id is a date followed by a 3-digit zero-padded ordinal number.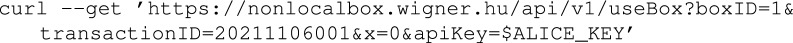 API output: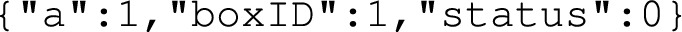 The box has emitted the reply $a=1$. The zero status implies that there is no error.
2. Let now Bob send $y=0$. Note that for $x=y=0$ the results should be correlated, so Bob should get $b=1$. And indeed,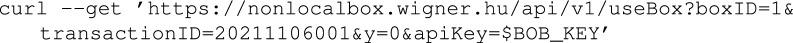 API output:
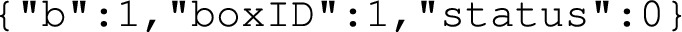
3. In a next transaction (with an incremented ID), Bob will be the first to send $y=1$: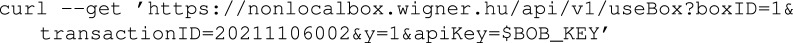 API output: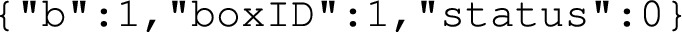 The box gave $b=1$.
4. Now assume that Alice also opts for $x=1$, thus the results should be anticorrelated, i.e. $a=0$ should be obtained. And indeed: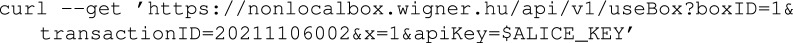 API output:
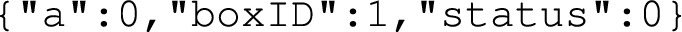

Note that both parties obtain a uniformly distributed random result for their inputs, when observed just locally. However, when analyzed together, the expected joint conditional probability of the Popescu-Rohrlich nonlocal box can be observed.

To demonstrate and verify this we have performed a systematic test of the API; a virtual Bell-experiment. The code of the test is available so that the test can be reproduced; the documentation of the test contains all technical details. To run the test, a pair of API keys is needed, it assumes to be run by two users, one playing the role of Alice, the other that of Bob. At the time of writing the test code supports two-input-two-output boxes. We have tested on a PR-box but the same can be done with any other of these.

The test can be run as follows. In a preparation phase, Alice and Bob create a box, e.g. a Popescu–Rohrlich box to be tested. They agree on the box ID. In the first phase of the test each party runs a program which carries out a number of “experiments” consisting of a number of transactions. The tests can certainly be run on separate computers. Within the measurement, a sequence of transaction IDs is generated. The transactions are, however, executed in a random order different at each party in order to verify the asynchronous operation. In each transaction, both parties generate a random input bit locally, and obtain the output from the API. The results of these “measurements” are saved into files. In the evaluation phase the saved test results are collected to the same computer and the empirical joint conditional probability distributions are evaluated. The empirical probability distribution should agree with the expected behavior.

We have carried out such a test to verify the proper operation of the simulation. In particular we have verified the operation of a PR-box whose behavior (i.e. theoretical conditional probability distribution) is tabulated in Table 3. In Table 4 we present the result of 5 experiments, with 40,000 measurements each. The inputs at the measurements are uniformly distributed both on Alice’s and Bob’s side, hence, in each experiment there are about 10, 000 samples for each distribution.

We have found that the events with zero probability according to theoretical conditional probability distribution never occur in the samples, which is not unexpected: it should be so by the construction of the algorithm generating the data. Once the input pair (*x*, *y*) is given, there are two possible outcomes remaining with equal probabilities, hence we are testing whether the respective part of the sample is drawn according to a Bernoulli-distribution with equal probability of the two events. In fact the algorithm generates the respective random bits directly, hence the present test is essentially a direct test of the random generator used as a source of random bits in our implementation.

The empirical distributions, i.e. the relative frequencies of the outcome pairs are apparently close to the uniform distribution. In order to quantitatively verify whether the API realizes the expected random behavior, we apply a standard $\chi ^2$ statistical test using the implementation in the Python SciPy package ^29^ for each (*x*, *y*) input pair in each experiment. The test yields the p-value, a parameter between 0 and 1. It is commonly accepted that if this parameter is in the range [0.05, 0.95] then the test is passed: the results are really random and the distribution really belongs to the equal probability of the two events.

The zero probability events never occur, in complete agreement with the theoretical distribution, the $\chi ^2$ test should be restricted to the support of the probability distribution. The number of samples is set to a high value as the $\chi ^2$ test is better done on large samples. (The empirical distribution is similar to the presented one already after drawing a few hundred samples, but it does not yet prove the appropriate behavior in a statistical sense.) The data of Table 4 convincingly prove that the API works as expected. We have published the code ^30^ implementing the whole testing process, including the creation of the box, the experiment, and the evaluation in the form of scripts.

**Table 4:** Result of 5 experiments with a Popescu–Rohrlich box, with 40, 000 measurements (transactions) in each experiment.

Exp.	*x*	*y*	*N*	$q_{00}$	$q_{01}$	$q_{10}$	$q_{11}$	p-value
1	0	0	10172	0.5010	0.0000	0.0000	0.4990	0.8428
1	0	1	9855	0.4902	0.0000	0.0000	0.5098	0.0519
1	1	0	10062	0.4926	0.0000	0.0000	0.5074	0.1401
1	1	1	9911	0.0000	0.5046	0.4954	0.0000	0.3607
2	0	0	9902	0.4969	0.0000	0.0000	0.5031	0.5332
2	0	1	10025	0.4950	0.0000	0.0000	0.5050	0.3131
2	1	0	9902	0.5034	0.0000	0.0000	0.4966	0.4944
2	1	1	10171	0.0000	0.5009	0.4991	0.0000	0.8506
3	0	0	10046	0.5062	0.0000	0.0000	0.4938	0.2160
3	0	1	9947	0.5073	0.0000	0.0000	0.4927	0.1460
3	1	0	10034	0.4909	0.0000	0.0000	0.5091	0.0692
3	1	1	9973	0.0000	0.4921	0.5079	0.0000	0.1159
4	0	0	10188	0.4986	0.0000	0.0000	0.5014	0.7815
4	0	1	9963	0.4959	0.0000	0.0000	0.5041	0.4171
4	1	0	9984	0.5045	0.0000	0.0000	0.4955	0.3677
4	1	1	9865	0.0000	0.5036	0.4964	0.0000	0.4747
5	0	0	9989	0.4986	0.0000	0.0000	0.5014	0.7870
5	0	1	10156	0.4981	0.0000	0.0000	0.5019	0.7061
5	1	0	9909	0.5066	0.0000	0.0000	0.4934	0.1882
5	1	1	9946	0.0000	0.4964	0.5036	0.0000	0.4703

The first column is the ordinal number of the experiments, the second two are Alice’s and Bob’s input respectively. The next column is the number of transactions with this input pair. The next four columns contain the empirical probability distribution *q*. The last column is the p-value of the $\chi ^2$ test on the support of the probability distributions. Note that the events displayed with 0.0000 probability are the events with zero probability in the theoretical distribution, and indeed they never happened in any of the experiments.

## Conclusions

We have reported on the design and implementation of a RESTful WEB API service that implements nonlocal no-signaling correlations logically. Thereby it is capable of emulating nonlocal quantum correlations that are perhaps the most intriguing features of quantum mechanics and are essential ingredients of most applications in quantum information and communication, notably in device independent quantum cryptography. The described web service has also been implemented by us and we made it available to the community ^28^.

From the point of view of scientific research, one of our contributions is the algorithm that implements no-signaling correlations using a central trusted resource: we have not seen it before in the literature. The discussion of the asynchronous nature of no-signaling correlations can also be considered as a minor contribution of this kind. While it is mentioned in some previous contributions, probably because of its difficult implementation in quantum experiments, it gained less attention before. During the development we report here, this has lead us to finding the first known application in which the non-sequential use of nonlocal no-signaling resources is useful ^21^.

From the technological point of view our contribution makes nonlocal no-signaling correlations readily available using the perhaps most commonly used web service technology. Recall that trusted elements are involved also in practical quantum key distribution. Secure application entities, for instance, receive quantum keys from key management entities via RESTful APIs according to the ETSI-014 standard ^20^; all these elements are all considered as trusted. As opposed to that, our setup does not enable the remote parties to set up secure channels, and the implementation of the no-signaling correlations is based on the communication with a trusted server. If, however, the parties are not allowed to use any other means of communication to the server or any other party than the API calls, this alone will not enable them to build a working communication channel. The possible practical use of such a resource has not yet been considered. No-signaling correlations may find their use in the engineering of communication protocols: they can facilitate the coordination of actions without revealing the details of the decisions of parties. In addition, a service emulating quantum correlations can be used as a test and development environment for applications, even those designed for physical realizations of quantum correlations; device independent cryptographic protocols for instance. The API technology paves the way of designing a broad range of applications ranging from demonstrations on various platforms as well as practically useful ones, possibly.

Finally, from the dissemination point of view we believe that our API is an enabler in the experience-based teaching of nonclassical correlations and Bell-type quantum phenomena. At the time of writing of this paper, this application of our API is being tested in a high-school environment, and a mobile phone application is planned to increase the dissemination impact. We hope that these will help a number of people to understand the basics of the phenomena whose experimental study has lead to the Nobel prize in Physics awarded in 2022 ^31^.

## Data Availability

The datasets used and/or analysed during the current study available from the corresponding author on reasonable request.
